# Superior Vena Cava Syndrome in the Cancer Patient: A Case Study

**DOI:** 10.6004/jadpro.2012.3.6.4

**Published:** 2012-11-01

**Authors:** Elaine Kinnard

**Affiliations:** From Licking Memorial Health Systems, Newark, Ohio

## **ARTICLE** 

Patients with lung cancer are at risk for superior vena cava syndrome (SVCS). Here we present a case study of a gentleman with small cell lung cancer who develops a swollen neck, edema, and dyspnea upon exertion. Would you, as the advanced practitioner, recognize the problem?

Superior vena cava syndrome is a rare oncologic emergency that occurs in roughly 15,000 persons in the United States each year (Wilson, Detterbeck, & Yahalom, 2007). Superior vena cava syndrome has a distinct clinical presentation and can be life threatening. It is found in 3.8% of lung cancer patients at the time of diagnosis and more frequently associated with superior vena cava obstruction (Rowell & Gleeson, 2002). Superior vena cava syndrome is caused by cancer 95% of the time; the other 5% of cases could be related to thrombosis from insertion of venous catheters or pacemaker wires (National Cancer Institute, 2011). Characteristically, the onset of symptoms in SVCS occurs gradually over weeks to months (Parra, Rodas, Bartnik, & Puente, 2011).

## Pathophysiology

**Figure 1:**
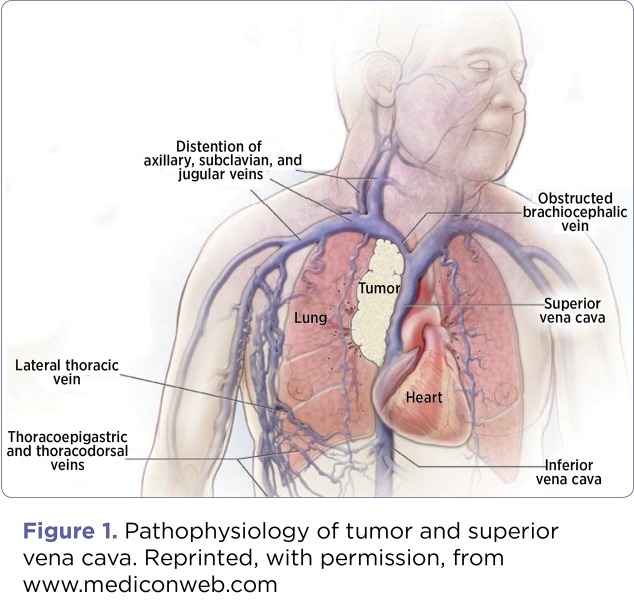
Figure 1. Pathophysiology of tumor and superior vena cava. Reprinted, with permission, from www.mediconweb.com

The superior vena cava (SVC) is a short large-diameter vein (about 7 cm long) that carries deoxygenated blood from the upper half of the body to the heart’s right atrium (Figure 1). The SVC is located in the anterior right superior mediastinum, surrounded by the sternum, ribs, vertebral bodies, and aorta (Nunnelee, 2007). When the SVC is compressed, blood flows through the collateral vascular network to create venous collaterals that dilate to accommodate blood flow. This results in the characteristic superficial blue vessels on the skin surface (Wilson et al., 2007). The obstruction causes distention of the axillary, subclavian, and jugular veins. The obstruction can increase edema in the luminal diameter of the pharynx and larynx, which causes the patient to develop stridor. Cerebral edema, which mayresult in headache and confusion, could occur and lead to cerebral ischemia and possible death (Lewis et al., 2011).

## Diagnosis

Superior vena cava syndrome consists of various symptoms due to compression of the SVC (Lepper et al., 2011). Early signs and symptoms include cough, dyspnea, hoarseness, chest pain, jugular vein distention, and edema of the hands, face, and/or neck. Family members may note these signs to be more prevalent in the early morning. More advanced signs and symptoms can be life threatening, such as respiratory distress, stridor, mental status changes, syncope, and cyanosis of the face and upper body (Colen, 2008).

Superior vena cava syndrome caused by cancer is a poor prognostic sign that is associated with high mortality. The advanced practitioner caring for a cancer patient with these symptoms needs to recognize the life-threatening diagnosis of SVCS as a priority consideration in the list of differential diagnoses. Additional differential diagnoses to be considered include pulmonary effusion, thrombosis, and catheter placement obstruction. The signs and symptoms of other possible diagnoses can be ruled out in the outpatient clinic if one understands and looks for the hallmarks of SVCS.

A thorough history and physical examination are critical, focusing on the signs and symptoms of SVCS. Advanced practitioners should specifically assess for dyspnea, upper extremity swelling, nonproductive cough, facial swelling, and collateral veins on the neck or upper chest and/or under the tongue (Nunnelee, 2007). A late symptom of this condition is Horner’s syndrome, identified by ptosis of the upper and lower eyelids, an ipsilateral constricted pupil, and/or loss of sweating on one side of the face (Aziz, 2010).

A chest CT with IV contrast or MRI can be performed to assist in evaluating the underlying pathology, which most likely will be tumor. The gold standard for evaluation of the SVC is a venogram (Lewis et al., 2011). The SVC diameter and length of the SVC stenosis/occlusion can be measured with these studies to help plan for endovascular treatment if warranted (Lepper et al., 2011).

## Treatment

Because SVCS is a life-threatening emergency, early diagnosis and appropriate intervention are imperative. Treatment for SVCS depends on the cause and prognosis of the underlying disease (Battal et al., 2009). The goal of managing SVCS in malignancy cases is to relieve symptoms and treat the underlying causes (Sommers, 2012). If the cancer prognosis for the patient is poor, then it would be appropriate to relieve symptoms and offer comfort measures. In most cases where SVCS is caused by a malignancy, chemotherapy or radiation is the treatment of choice (Cho, Janho, & Mohan, 2011). Vascular thrombosis may require thrombolytic agents. Endovascular management is another well-recognized treatment for malignant SVCS. A stent is placed in the SVC to increase the blood flow through the stenosis (Cho et al., 2011).

There are some non–evidence-based treatments for SVCS available. The use of steroids and diuretics is still being studied, yet so far they have not been shown to be effective in reducing symptoms (Lewis et al., 2011).

## Conclusion

Superior vena cava syndrome occurs infrequently, but it can be a life-threatening oncologic emergency. The advanced practitioner needs to be aware of patients at high risk for this condition. Early recognition of signs and symptoms of impending SVCS is essential in an ambulatory setting. Accurate diagnosis and aggressive management of the underlying cause of the SVCS (tumor or clot) should be timely and are imperative for patients to achieve positive outcomes. Randomized controlled trials are lacking for most treatment options seeking to directly treat the signs and symptoms of SVCS (Lepper et al., 2011).

##  Case Study

**Figure 2:**
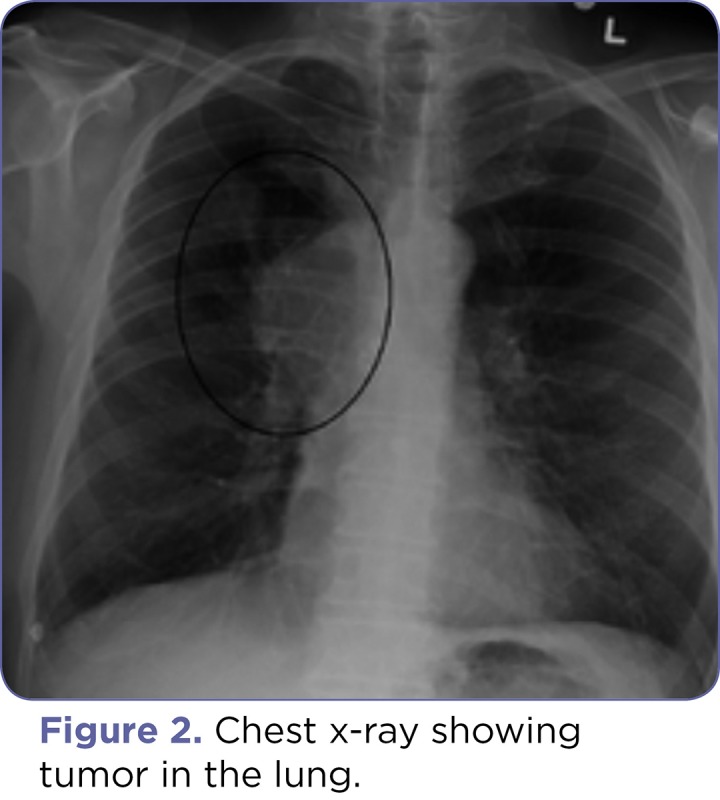
Figure 2. Chest x-ray showing tumor in the lung.

Mr. P. is a 67-year-old male who was originally seen by the pulmonologist after a chest x-ray revealed a right upper-lobe lung mass (Figure 2). Bronchoscopy revealed an extensive endobronchial tumor, and a biopsy was positive for small cell lung cancer. The patient was referred to the oncologist.3

**Figure 3:**
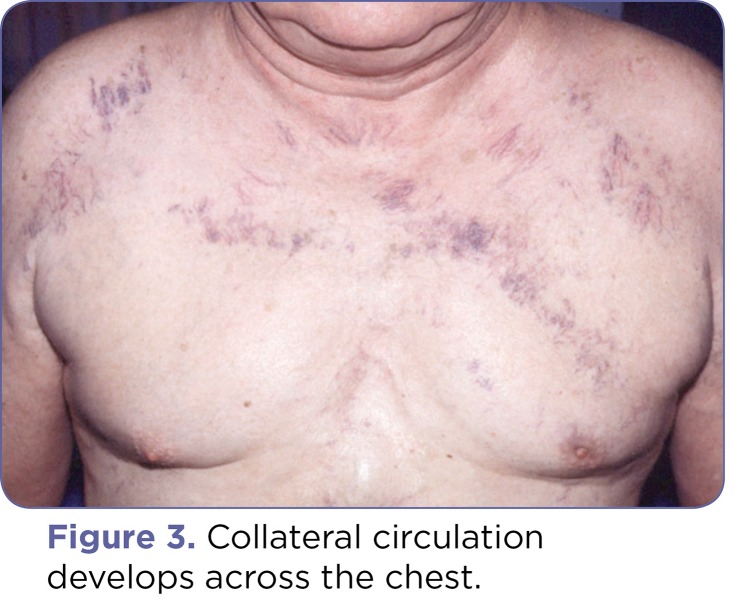
Figure 3. Collateral circulation develops across the chest.

During the first oncology office visit, Mr. P. complained of increasing fatigue. He stated, "I become short of breath when I bend over. It lasts about 60 seconds, with some dizziness and head pain." Review of systems is positive for neck swelling noted in the morning, hoarseness of voice during the past week, purplish discoloration across his chest (Figure 3), thick vessels under his tongue, increased dyspnea on exertion, difficulty swallowing, and a dry cough lasting over a week. Pertinent physical findings are as follows: blood pressure 124/64, respirations 20, pulse 72 regular, temperature 97.8°; diffuse edema in the neck; dilated, engorged blood vessels on the chest and under the tongue; and edema in the left arm and hand.

Mr. P.’s current complaint is dyspnea, a sudden onset of a swollen neck, and a new diagnosis of small cell lung cancer. Because his symptoms are suggestive of superior vena cava syndrome (SVCS), which is potentially life threatening, he was immediately admitted to the hospital. A CT scan identified the source of the SVCS to be his malignancy. A CT angiogram also showed almost complete obstruction of the superior vena cava. The advanced practitioner’s admission orders included elevation of the head of the bed. In consultation with the collaborating physician, chemotherapy was immediately started. Mr. P. was in the hospital for 3 days as his symptoms responded quickly to the chemotherapy and he was able to breathe more easily. He was followed as an outpatient in the oncologist’s office weekly for labwork and received chemotherapy every 
